# Cochlear Implant Stimulation Parameters Play a Key Role in Reducing Facial Nerve Stimulation

**DOI:** 10.3390/jcm12196194

**Published:** 2023-09-25

**Authors:** Lutz Gärtner, Bradford C. Backus, Nicolas Le Goff, Anika Morgenstern, Thomas Lenarz, Andreas Büchner

**Affiliations:** 1 Department of Otolaryngology, Hannover Medical School, 30625 Hannover, Germany; gaertner.lutz@mh-hannover.de (L.G.); morgenstern.anika@mh-hannover.de (A.M.); lenarz.thomas@mh-hannover.de (T.L.); 2 Oticon Medical, 06220 Vallauris, France; brba@oticonmedical.com (B.C.B.); nile@oticonmedical.com (N.L.G.); 3 Cluster of Excellence “Hearing4all”, 30625 Hannover, Germany

**Keywords:** FNS, facial nerve, CI, cochlear implant, electrode, stimulation parameters, OMRP, Oticon Medical Research Platform

## Abstract

A percentage (i.e., 5.6%) of Cochlear Implant (CI) users reportedly experience unwanted facial nerve stimulation (FNS). For some, the effort to control this problem results in changing stimulation parameters, thereby reducing their hearing performance. For others, the only viable solution is to deactivate the CI completely. A growing body of evidence in the form of case reports suggests that undesired FNS can be effectively addressed through re-implantation with an Oticon Medical (OM) Neuro-Zti implant. However, the root of this benefit is still unknown: is it due to surgical adjustments, such as varied array geometries and/or positioning, or does it stem from differences in stimulation parameters and/or grounding? The OM device exhibits two distinct features: (1) unique stimulation parameters, including anodic leading pulses and loudness controlled by pulse duration—not current—resulting in lower overall current amplitudes; and (2) unconventional grounding, including both passive (capacitive) discharge, which creates a pseudo-monophasic pulse shape, and a ‘distributed-all-polar’ (DAP) grounding scheme, which is thought to reduce current spread. Unfortunately, case reports alone cannot distinguish between surgical factors and these implant-related ones. In this paper, we present a novel follow-up study of two CI subjects who previously experienced FNS before re-implantation with Neuro-Zti implants. We used the Oticon Medical Research Platform (OMRP) to stimulate a single electrode in each subject in two ways: (1) with traditional monopolar biphasic cathodic-first pulses, and (2) with distinct OM clinical stimulation. We progressively increased the stimulation intensity until FNS occurred or the sound became excessively loud. Non-auditory/FNS sensations were observed with the traditional stimulation but not with the OM clinical one. This provides the first direct evidence demonstrating that stimulation parameters and/or grounding—not surgical factors—play a key role in mitigating FNS.

## 1. Introduction

Cochlear implants (CIs) are the most successful sensory prosthetic devices developed to date and have revolutionized the world of audiology, offering hope to individuals with severe to profound hearing loss [1,2,3]. However, while CIs significantly improve auditory perception for many, the technology is not without its challenges. One such issue is the unwanted stimulation of the facial nerve (FNS), a side effect reported in an estimated 5.6% of CI users [4]. FNS can lead to involuntary facial twitching, vertigo, or indistinct pain and thereby impact quality of life. To unlock the benefits of CIs for these individuals, we need to gain a better understanding of the contributing factors that lead to FNS and circumvent them.

Traditionally, FNS in CI users has been managed through the adjustment of stimulation parameters by the audiologist. The most common type of stimulation for cochlear implants consists of biphasic pulses with a leading cathodic phase—a method derived from animal studies, which suggested its superior effectiveness [5,6]. These biphasic pulses are then amplitude modulated based on the time-varying envelope output from the filter linked to the corresponding electrode. The majority of CI manufacturers employ this paradigm. For a more comprehensive overview of CI functionality, readers are directed to [3]. To prevent unpleasant facial nerve stimulation, clinicians often lower the pulse current (and expand the pulse duration) or deactivate troublesome electrodes altogether. However, these methods do not always work, and even when they do, they can compromise CI performance. In severe cases, the CI becomes unusable.

Some CI manufacturers, such as MED-EL (Innsbruck, Austria), offer a ‘triphasic’ stimulation mode. In this mode, there are 3 pulse phases: (1) a leading cathodic pulse phase; (2) an intermediate anodic phase presented with twice the phase duration; and (3) a final repeated cathodic phase. While this method has shown effectiveness in reducing unwanted FNS for some CI patients [5,6], it does not always work and usually results in reduced battery life.

Emerging evidence from case reports suggests a promising treatment for severe cases is re-implantation with the Oticon Medical (OM) (Smørumnedre, Denmark) Neuro-Zti implant. Re-implantation with this device has been shown to effectively address FNS. However, the reason for this is not yet fully understood. While surgical adjustments such as varying array geometries and positioning may play a role, there are also distinct implant-related attributes to consider. These include the unique stimulation parameters used by OM devices, such as anodic leading pulses, passive capacitive charge return, and their unconventional distributed all-polar (DAP) grounding scheme [7]. Both elements are distinct from other CI systems. Moreover, in the OM device, loudness is not coded by pulse current but rather by pulse duration. Understanding whether the reduced FNS seen in the literature stems from surgical factors or from these implant-related ones will provide valuable insight to further improve CI technology and enhance patient outcomes and quality of life for those suffering from FNS.

This analysis presents a novel follow-up study on two CI subjects who experienced FNS prior to re-implantation with OM Neuro-Zti implants [8] and reveals the reasons why this intervention helped them.

## 2. Materials and Methods

### 2.1. Subjects

Two subjects who had previously suffered from FNS during CI stimulation and had been re-implanted with Oticon Medical Neuro-Zti devices [8] were further investigated during a clinical follow-up visit. One of these patients was initially fitted with Advanced Bionics (Valencia, CA, USA) HiRes Ultra 3D implants and a mid-scala electrode in both ears. The other patient had originally been fitted with a MED-EL SYNCHRONY implant with a FLEX28 electrode in one ear.

Our goal was to gain deeper insight into the subjects’ behavioral perceptions of different stimuli, with the ultimate aim of further improving their clinical outcomes. 

### 2.2. Stimuli

**Figure 1:**
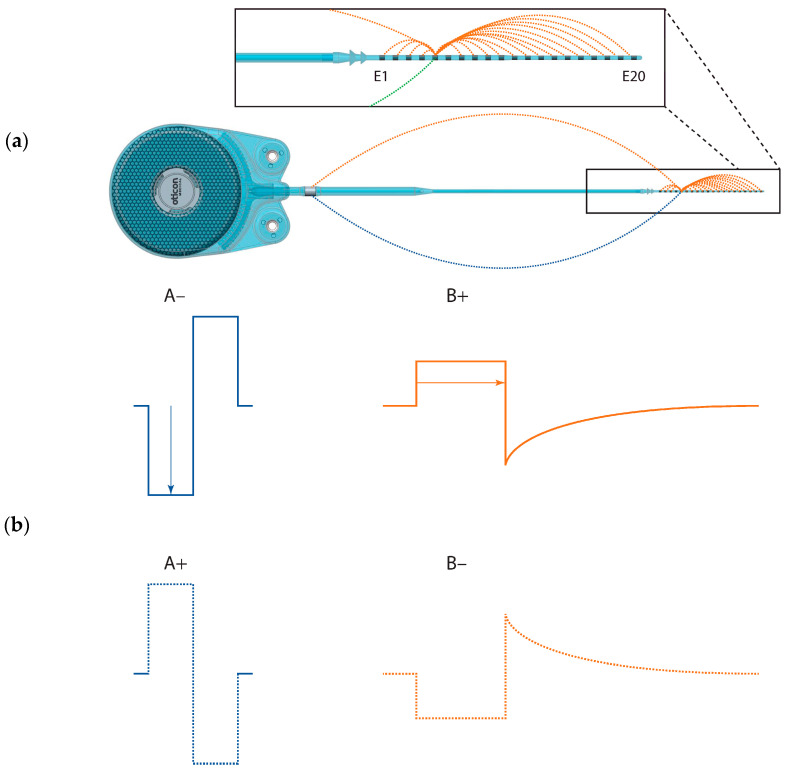
Panel (**a**) shows a diagram of the Neuro-Zti implant illustrating current paths (dotted lines) for monopolar (MP) grounding used for stimuli A− and A+ (blue) vs. distributed-all-polar (DAP) grounding used for B+ and B− (orange). It also shows the electrode positions and numbering. Panel (**b**) shows sketches of the pulse shapes used in the experiment for MP grounding (blue) and DAP grounding (orange). The first row shows the clinical pulses in each case, with arrows denoting clinical loudness coding; the second row shows the reversed polarity pulses used with Subject S2.

We used the Oticon Medical Research Platform (OMRP) [9] to directly stimulate single electrodes on the Neuro-Zti Implant in two ways: (1) using stimulus parameters designed to replicate those of their prior CI, and (2) using the parameters of their current clinical CI mode. With Subject S2, we also investigated reversing the polarity of these pulses for a total of 4 stimulus types (Table 1 and Figure 1). All stimuli were presented in a pulse train with a 50% duty cycle (500 ms on and 500 ms off) using pulses presented at a rate of 500 Hz.

In each trial, we gradually increased the stimulation charge (i.e., pulse duration or amplitude) until either (1) the sound was too loud or (2) non-auditory sensations became too intense for the subject.

### 2.3. Procedure

Testing was conducted during a scheduled clinical fitting session. The session was divided into three parts, each lasting about 30 min, interspersed with 5-min breaks. The total testing time was approximately 2 h.

**Figure 2:**
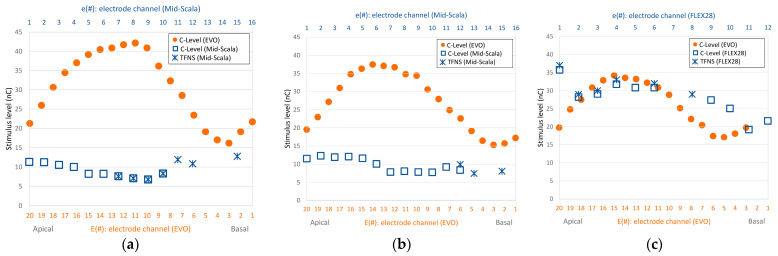
Selected results from case reports presented in [8]. C-level before and about half a year after re-implantation with Oticon Medical Neuro Zti EVO. Asterisks refer to the FNS thresholds with the previously implanted electrode. The upper x-axis refers to the channel number (#) of the previously implanted electrode; the lower x-axis refers to those of the EVO electrode. (**a**) Subject S1, right; (**b**) Subject S1, left; (**c**) Subject S2, right.

In the first session, we selected suitable electrodes for the experiment from the regions where subjects had previously reported strong FNS responses (Figure 2). We conducted a search to determine which electrode to use for each subject utilizing the ‘A−’ stimulus (Table 1). This was chosen to mimic their previous cochlear implant and thereby give a good chance of eliciting an FNS response. For the search, we incrementally increased the pulse charge until either the subject reported a non-auditory sensation or the loudness reached an uncomfortable level. Not all electrodes that previously caused FNS (Figure 2) induced FNS responses with the Oticon Medical implant. Those that did, did so at different charge levels than previously reported (higher ones for subject S1, and lower ones for subject S2). In the end, we selected the electrode that elicited the most substantial FNS response to study in detail.

In the two following sessions, we stimulated the chosen electrode using either (1) the current clinical mode of their Oticon Medical CI (stimulus B+) or (2) a non-clinical mode designed to mimic their previous CI (stimulus A−). This allowed us to directly compare the in-situ effect of these stimulation modes while keeping implant hardware and subject factors consistent. For each stimulation mode, we gradually increased the intensity. At each charge level, we asked the subjects to: (1) rate the loudness on a scale from 0 (inaudible) to 10 (very loud); (2) describe any non-auditory sensations qualitatively; and (3) indicate whether they were comfortable enough to continue. Due to time constraints, we did not interleave testing modes; this would have necessitated reconfiguring the implant between presentations. 

For subject S1, only stimuli of type A− and B+ were explored as described above, because it took some time until we found an electrode that exhibited strong non-auditory side effects. For all stimuli presented to subject S1, charge was increased using pulse duration due to a software limitation (Table 1). Fortunately, for subject S2, the software limitation was overcome, and we then used current coding for stimuli A−/A+ and duration coding for stimuli B−/B+ as would have been carried out clinically. We also had time to explore the effect of reversing pulse polarity for subject S2 in session 3 (Figure 1).

## 3. Results

**Figure 3:**
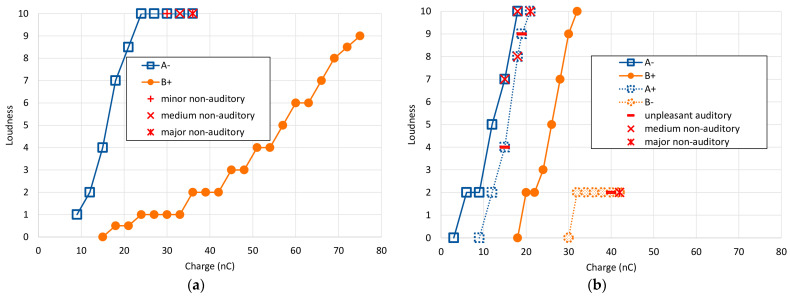
(**a**) Subject-reported loudness vs. individual pulse charge delivered for subject S1 (left electrode E5) showed faster loudness growth with stimulus A− than with B+. Stimulus A− also elicited non-auditory FNS sensations. (**b**) Subject S2 (right electrode E18) reported similar slopes but rightward-shifted loudness growth functions between A− and B+. Non-auditory sensations on both panels show how stimulus A− led to FNS stimulation at lower charge levels than for other stimulus types and how non-auditory sensations were affected in a markedly different way from the loudness percepts. Using stimulus B−, we were unable to achieve sufficient loudness. Instead, pronounced side effects were observed.

The results of the main single electrode experiments for both subjects across all stimulus types are shown in Figure 3 as loudness growth curves. Reported non-auditory/FNS sensations are overlayed as separate symbols. For both subjects, the charge required to reach equivalent loudness levels was higher for stimulus B+ than for A− by more than a factor of 2. However, the growth in loudness was very different between the two. Subject S1 exhibited a rightward shift (~20 nC) and a slower loudness growth for the B+ stimulus, while Subject S2 only exhibited the rightward shift.

Reversing the polarity highlighted how non-auditory sensations were affected in a markedly different way than the loudness percept. More detailed results for each subject are presented individually below. 

### 3.1. Subject S1

Using stimulus type A−, first the right ear was tested for side effects. On electrodes E3 and E7, the patient felt some vibration in the outer ear at very loud auditory perception (loudness 10, charge level 36 nC (E3) and 27 nC (E7)). On electrode E11, no non-auditory sensation was reported up to a charge level of 33 nC (loudness = 10). These three electrodes correspond approximately to electrodes e14, e11, and e8 of the previous CI (see Figure 2a), where severe FNS had previously been perceived at soft loudness levels. Thereafter, measurements were performed on the left side on electrodes E7 and E16, again without any non-auditory sensation (loudness 10, charge level 27 nC (E7) and 33 nC (E16). These electrodes matched approximately electrodes e11 and e4 of the previous CI (see Figure 2b).

Finally, measurements on electrode E5 (left) unveiled certain non-auditory side effects. Specifically, a peculiar sensation was reported at a charge level of 30 nC (perceived loudness level at 10). When the charge was increased to 33 nC, the subject described feeling a vibration in the outer ear (see Table 2 for a summary of these findings). Further, at a charge level of 36 nC, this sensation was accompanied by a facial tingle. At this point, a stapedial reflex was objectively confirmed using tympanometry, and we discovered that the reflex was elicited at charge levels from 30 nC upwards, matching the subject’s reported perceptions. We selected this E5 electrode for our further investigation.

While the type A− stimulus presented a rather rapid increase in loudness perception, type B+ exhibited a shallower growth and did not induce any non-auditory side effects (Figure 3a and Table 3). With the use of type B+ stimulation, the loudness level did not exceed 9, and even at charge levels of 75 nC—the maximum possible in our setup—the stapedial reflex was not elicited. 

### 3.2. Subject S2

The search for electrodes with side effects began with electrode E18, which was approximately equivalent to electrode e2 of the previous implant. This was where a comfortably loud stimulation level had previously evoked FNS (refer to Figure 2c). Using the A− stimulus, the patient experienced vertigo at 15 nC with a loudness level of 7. When the charge was increased to 18 nC, the patient reacted and described a mild but discomforting sensation akin to ‘pain in the head’ and a loudness level of 10 (Table 4). We selected this E18 electrode for our further investigation.

With the anodic-leading, passive-discharge B+ stimulus, the patient reported no non-auditory side effects, even up to a charge level of 32 nC (loudness 10). When testing with the reversed polarity using stimulus A+, the patient described an unpleasant buzzing sensation at 15 nC (perceived loudness at level 4). By 18 nC, the subject experienced a sensation akin to ‘a force pulling on the head’ and rated the loudness at level 8. The buzzing persisted at 19 nC with a loudness perception of level 9. By the time stimulation reached 21 nC (loudness level 10), nystagmus was observed—indicative of an activation of the vestibular system due to the electrical stimulation.

In contrast, with cathodic-leading passive-discharge stimulation (B−), there was only a modest rise in perceived loudness, saturating at a level 2 perception between 32 and 42 nC with no additional growth. At 40 nC, the subject described a deterioration in auditory quality. By 42 nC, the sensations reported were uncomfortably familiar to those previously experienced with her prior cochlear implant, characterized by a blend of vague pain and dizziness. All loudness growth functions for subject S2 are shown in Figure 3b, and corresponding data is listed in Table 5.

## 4. Discussion

Approximately 5.6% of all cochlear implant users report experiencing aberrant facial nerve stimulation (FNS) as a side effect of their CI implantation [4]. For users presenting with FNS, audiologists may first attempt to control the problem by re-programming the device to produce lower currents, followed by turning off offending electrodes—both of which can reduce speech comprehension [4]. If these solutions fail, clinics have observed that re-implantation with an Oticon Medical Neuro Zti implant can resolve FNS issues [8,10,11,12,13]. Indeed, for our two subjects, re-implantation with the Oticon Medical device not only completely resolved FNS but also improved speech recognition [8].

The mechanisms that underlie this improvement are not yet well understood. However, we can reasonably expect that factors that affect the local electrical fields near neural activation points for the auditory and facial nerves—combined with how those nerves respond to these fields—are involved. These factors include: (1) electrode proximity; (2) factors that affect current spread (e.g., grounding and pulse duration and shape); and (3) polarity. In the subsequent four sections, we explore these factors and discuss the distinct characteristics of the Oticon Medical device in these areas. It is important to clarify that our intention is to shed light on these differences and not to imply that these distinctions inherently make the Oticon Medical device superior to others.

### 4.1. Electrode Proximity

The proximity between the stimulating electrodes and the neural activation sites of the auditory nerve fibers (ANF) is influenced by the type of electrode array used. Common understanding suggests that modiolus-hugging or mid-scala arrays might offer advantages in minimizing FNS over the lateral wall array design, used—among others—in the Oticon Medical electrode array. Case studies, such as Battmer et al. [14], indicate that electrodes positioned closer to the ANF require less current for excitation. This reduced current potentially leads to limited current spread, decreasing the likelihood of stimulating more distant non-auditory neural structures. Indeed, when looking across the literature, electrode array type does emerge as a statistically significant factor [4]. However, other case studies—including those of the two subjects in this manuscript—demonstrate that the Oticon Medical device is effective in alleviating unwanted FNS. Consequently, as we’ve previously argued [8], stimulation-related factors likely have a larger impact.

### 4.2. Grounding

Beyond geometry, other factors determining the current spread are also relevant when considering the activation of more distant, non-auditory neural structures. A notable distinction between the OM devices and others lies in their DAP grounding scheme (see Figure 1). With DAP, approximately 80% of the current returns to intra-cochlear electrodes and the remaining 20% to an extra-cochlear electrode [7]. By contrast, conventional MP-grounding returns all the current through the extra-cochlear electrode. This MP grounding mechanism theoretically results in a broader dispersion of the overall electrical field, making it more likely to intersect with the facial nerve.

In addition to DAP and MP, ‘bipolar’ and ‘common ground’ schemes are also in clinical use, with the latter commonly observed in older Cochlear^®^ (Cochlear Limited, Sydney, Australia) devices. Both return current via intracochlear electrodes. A study investigating the effects of different grounding strategies on FNS efficacy, conducted using 204 electrically evoked compound action potential (eCAP) input/output functions recorded from 33 ears of 26 guinea pigs, revealed that—for biphasic pulses—the broad-MP grounding was associated with a high occurrence of FNS (65%), while bipolar and an experimental tripolar configuration (expected to be the most focused) generated only 20% and 2% of FNS occurrences, respectively [15].

### 4.3. Pulse Duration and Shape

Altering the grounding scheme modifies the spatial distribution of current. While certain configurations might reduce this spread, predicting current pathways in individual anatomies is challenging. Specific grounding configurations, like bipolar or multipolar schemes—which are presumed to be more focused—typically require higher charge levels to reach equivalent loudness percepts vs. MP grounding [12]. This could, in turn, lead to a broader current spread again.

OM differs from most CI manufacturers in its encoding of loudness; it uses pulse duration rather than current amplitude. Consequently, the current is consistently set at a relatively low level, even for intense sounds. It has been shown that this approach can lead to a more focused area of excitation, especially at higher stimulation levels [16]. 

The OM pulse shape is also different than the standard biphasic one. It begins with an active rectangular phase, but rather than being followed by a symmetric shape, the charge return is via passive (capacitive) discharge, leading to an exponential decay (Figure 1). This unique pulse waveform requires only half the stimulation power needed for generating symmetric biphasic pulses. While the amplitude of the second phase varies based on the duration and current of the initial active phase, it is typically much smaller, creating a pulse shape akin to a pseudo-monophasic one. Such pseudo-monophasic (or asymmetric) pulses are known to be charge-efficient, activating nerve fibers with lower charge levels than symmetric biphasic pulses [17,18]. Mathematical modeling by Frijns et al. [19] also suggests that asymmetric pulses like these might act to reduce current spread to some extent compared to their symmetric counterparts.

In essence, employing pulse duration coding and pseudo-monophasic pulse shapes appears to limit current spread within the cochlea, potentially decreasing the likelihood of FNS.

### 4.4. Pulse Polarity

Not only does the OM device have a unique grounding scheme and pulse shape, but it also has opposite polarity to the standard clinical biphasic pulses. The majority of CI manufacturers initiate their biphasic pulses with a cathodic phase, while OM devices begin with an anodic phase. This alternative polarity, combined with the pseudo-monophasic pulse shape, seems to significantly impact the occurrence of FNS, as observed in our two subjects. Specifically, when using the OM’s clinical pulses, both subjects experienced no side effects. However, when traditional biphasic active stimuli with MP grounding were applied, side effects were evident. To better understand the effect of the polarity alone, we inverted the polarity of the stimuli in the OM stimulation mode for subject S2. We found that those pseudo-monophasic cathodic-leading pulses (using DAP grounding) did induce FNS, even at low loudness levels and in the absence of any auditory loudness growth.

Notably, when using the clinical anodic leading pulses, the subject reached her maximum tolerable loudness (level 10) at a charge of 32 nC without any side effects. However, when cathodic-leading pulses of the same type were used, reported loudness plateaued at level 2, while side effects continued to escalate as charge levels were increased. This striking contrast between these two conditions suggests that, for this subject, anodic stimulation primarily excited auditory nerve structures, while cathodic stimulation was effective at activating other neural structures, such as the facial nerve. 

### 4.5. Summary and Further Considerations

For our two subjects utilizing the OM Neuro ZTI implant, we observed that traditional symmetric biphasic cathodic-leading pulses in monopolar stimulation mode could elicit FNS. However, when using pseudo-monophasic anodic-leading pulses in DAP grounding mode—the clinical standard setting of the ZTI implant—it was impossible to trigger FNS even when raising charge levels at the subjects’ maximum tolerable loudness.

Conversely, and of significant note, we found that pseudo-monophasic cathodic-leading pulses in all-polar grounding mode could induce FNS at lower charge levels, while provoking auditory sensations required much higher charge, and even then, the auditory sensations were only soft. This striking contrast between these two conditions strongly suggests that anodic stimulation is primarily effective at exciting auditory nerve structures, while cathodic stimulation appears to predominantly activate other neural structures, such as the facial nerve.

Our findings, though just from two subjects, lend further support to an expanding body of research suggesting that the auditory and facial nerves exhibit differential sensitivity to electrical stimulation based on polarity [20,21,22,23,24]. However, unlike previous studies, which have primarily relied on action potential recordings in CI subjects elicited with active biphasic pulses, our study provides novel evidence from direct subjective feedback obtained from two human subjects using pseudo-monophasic pulses. We demonstrate that anodic currents are markedly more effective in selectively stimulating the neural structures associated with the auditory nerve while minimizing activation of the facial and other non-auditory neural structures. The hypothesis that the auditory nerve may be more sensitive to anodic stimulation while the facial nerve is more responsive to cathodic stimulation could also partially explain the reduced FNS symptoms observed with triphasic stimulation in the MED-EL device, which also uses a longer and presumably dominant anodic phase. Using an anodic-leading (or anodic dominant) pulse could, in theory, allow for more targeted stimulation of the auditory nerve, potentially reducing unwanted activation of other nerves like the facial nerve [25].

## 5. Conclusions

We conclude that CI stimulus parameters and grounding rather than surgical or electrode array changes were key factors in reducing FNS for our two subjects, and we suggest that this may hold true more generally. Our data indicates that the active anodic phase of the stimulus predominantly activates the auditory nerve fibers. In contrast, the cathodic phase seems more inclined to stimulate other neural structures, such as the facial nerve, leading to undesired side effects. Further untangling the relative contributions of polarity, pulse shape, pulse current vs. duration, and grounding to FNS will be a rich area for future investigations.

## Figures and Tables

**Table 1:** Stimulus parameters that were used during this study.

Parameter	A− (Clinical)	A+	B+ (Clinical)	B−
charge return	active	active	passive	passive
leading phase	cathodic	anodic	anodic	cathodic
pulse shape	biphasic	biphasic	pseudo- monophasic	pseudo- monophasic
grounding	MP	MP	DAP ^2^	DAP ^2^
loudness coding	current/duration ^1^	current/duration ^1^	duration	duration

^1^ Subject S1 used duration coding for stimuli A− and B+ (current = 0.6 mA). Subject S2 used duration coding for stimuli B−/B+ (current = 0.4 mA) and current coding for stimuli A+/A− (duration = 30 µs). Note: We improved the software between S1 and S2 to allow current coding. ^2^ Distributed-all-polar (DAP) [7] indicates that current returns via the case electrode, like MP (monopolar) grounding, and simultaneously to all non-stimulating intra-cochlear electrodes, sometimes called ‘common ground’ (CG) (Figure 1).

**Table 2:** Results of the search for electrodes with side effects. FNS thresholds taken from subject S1’s prior implantation (left and right) are compared with observations using the re-implanted OM device and a similar stimulation type (A−). The OM device exhibited higher FNS thresholds for this subject during the acute testing.

Prior Implant (Stimulus A−)		Neuro-Zti Implant (Stimulus A−)			
electrode number	FNS Threshold (nC)	Electrode number	Charge (nC)	Loudness	Observation
e14 (R)	14	E3 (R)	36	10	outer ear vibration
e11 (R)	12	E7 (R)	27	10	outer ear vibration
e8 (R)	8	E11 (R)	33	10	none
e11 (L)	10	E7 (L)	27	10	none
e4 (L)	N/A	E16 (L)	33	10	none
e13 (L)	8	E5 (L)	36	10	facial tingle

**Table 3:** Current and duration parameters used while testing subject S1 left ear electrode E5 using either stimulation style A− or B+ (Table 1) and the reported loudness (0 = unheard, 10 = very loud) perceptions for each of these. Comments and observations from the subject concerning any non-auditory sensations are highlighted with footnotes.

Stimulation	A−	B+
(fixed parameter)	(0.6 mA)	(0.6 mA)
Charge (nC)	Loudness	Loudness
9 *	1	
12	2	
15	4	0
18	7	0.5
21	8.5	0.5
24	10	1
27	10	1
30	10 ^1^	1
33	10 ^2^	1
36	10 ^3^	2
39		2
42		2
45		3
48		3
51		4
54		4
57		5
60		6
63		6
66		7
69		8
72		8.5
75		9

* The lowest possible value. ^1^ The subject reported ‘feeling’ something. ^2^ The subject reported an ‘outer ear vibration’. ^3^ The subject reported stronger ‘outer ear vibration’ and a facial tingle.

**Table 4:** Results of the search for electrodes with side effects. FNS thresholds taken from subject S2’s prior implantation (right) are compared with observations using the re-implanted OM device and a similar stimulation type (A−). For this subject, the OM device exhibited non-auditory thresholds at slightly lower charge levels during acute testing.

Prior Implant (Stimulus A−)		Neuro-Zti Implant (Stimulus A−)			
Electrode number	FNS Threshold (nC)	Electrode number	Charge (nC)	Loudness	Observation
e2	28	E18	15	7	vertigo
		E18	18	10	mild pain in the head

**Table 5:** Current and duration parameters used while testing subject S2 right ear electrode E18 using stimulation styles A−, A+, B−, or B+ (Table 1) and the associated reported loudness (0 = unheard and 10 = very loud) for each of these. Comments and observations from the subject concerning any non-auditory sensations are highlighted with footnotes.

Stimulation	A−	A+	B+	B−
(fixed parameter)	(30 µs)	(30 µs)	(0.4 mA)	(0.4 mA)
Charge (nC)	Loudness	Loudness	Loudness	Loudness
3	0			
6	2			
9	2	0		
12	5	2		
15	7 ^1^	4 ^3^		
18	10 ^2^	8 ^4^	0	
19		9 ^5^		
20			2	
21		10 ^6^		
22			2	
24			3	
26			5	
28			7	
30			9	0
32			10	2
34				2
36				2
38				2
40				2 ^7^
42				2 ^8^

^1^ The subject reported that the sound was ‘not unpleasantly loud’ but that it came with vertigo. ^2^ The subject winced and reported an unpleasant but mild ‘pain in the head’. ^3,5^ The subject reported an unpleasant buzzing sound. ^4^ The subject experienced a feeling similar to ‘a force pulling on her head’. ^6^ Observed nystagmus (involuntary eyes moving), a vestibular effect. ^7^ The subject reported deterioration of sound quality. ^8^ Unpleasant non-auditory sensations. The subject reported that this is ‘super unpleasant’ but still auditorily soft; the sensation reminded her of her previous CI.

## Data Availability

Data tables are included within the manuscript.
